# Supplementary figures and images for: Clinical Outcomes and Quality of Life after Patent Foramen Ovale (PFO) Closure in Patients with Stroke/Transient Ischemic Attack of Undetermined Cause and Other PFO-Associated Clinical Conditions: A Single-Center Experience
Source: J Clin Med. 2023 Sep 5;12(18):5788. doi: 10.3390/jcm12185788 (PMC10531865; doi:10.3390/jcm12185788)

## Slide 1
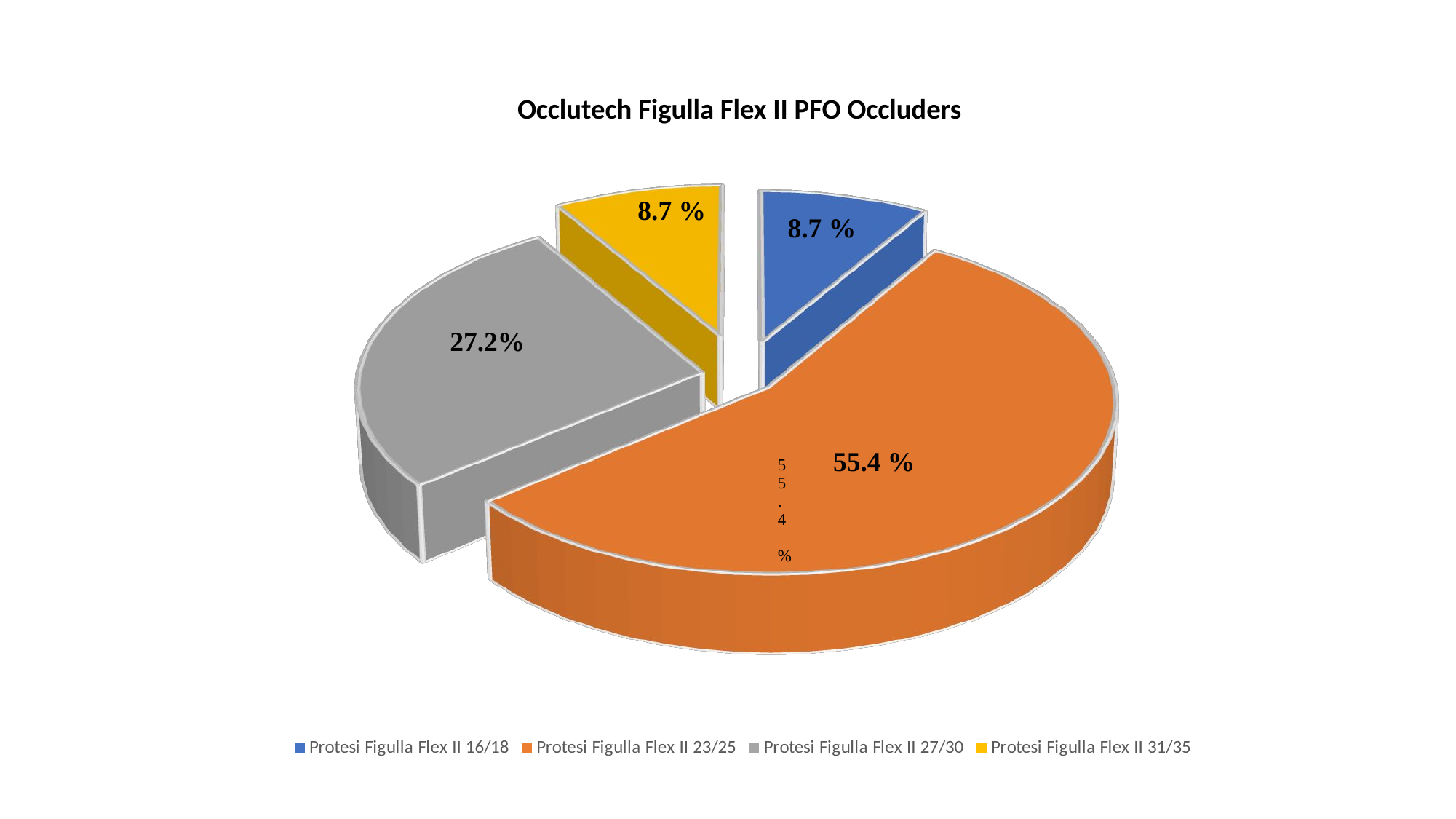

Occlutech Figulla Flex II PFO Occluders
[unsupported chart]

Supplement: Supplementary file 1 [file jcm-12-05788-s001.zip › Figure S1.pptx]

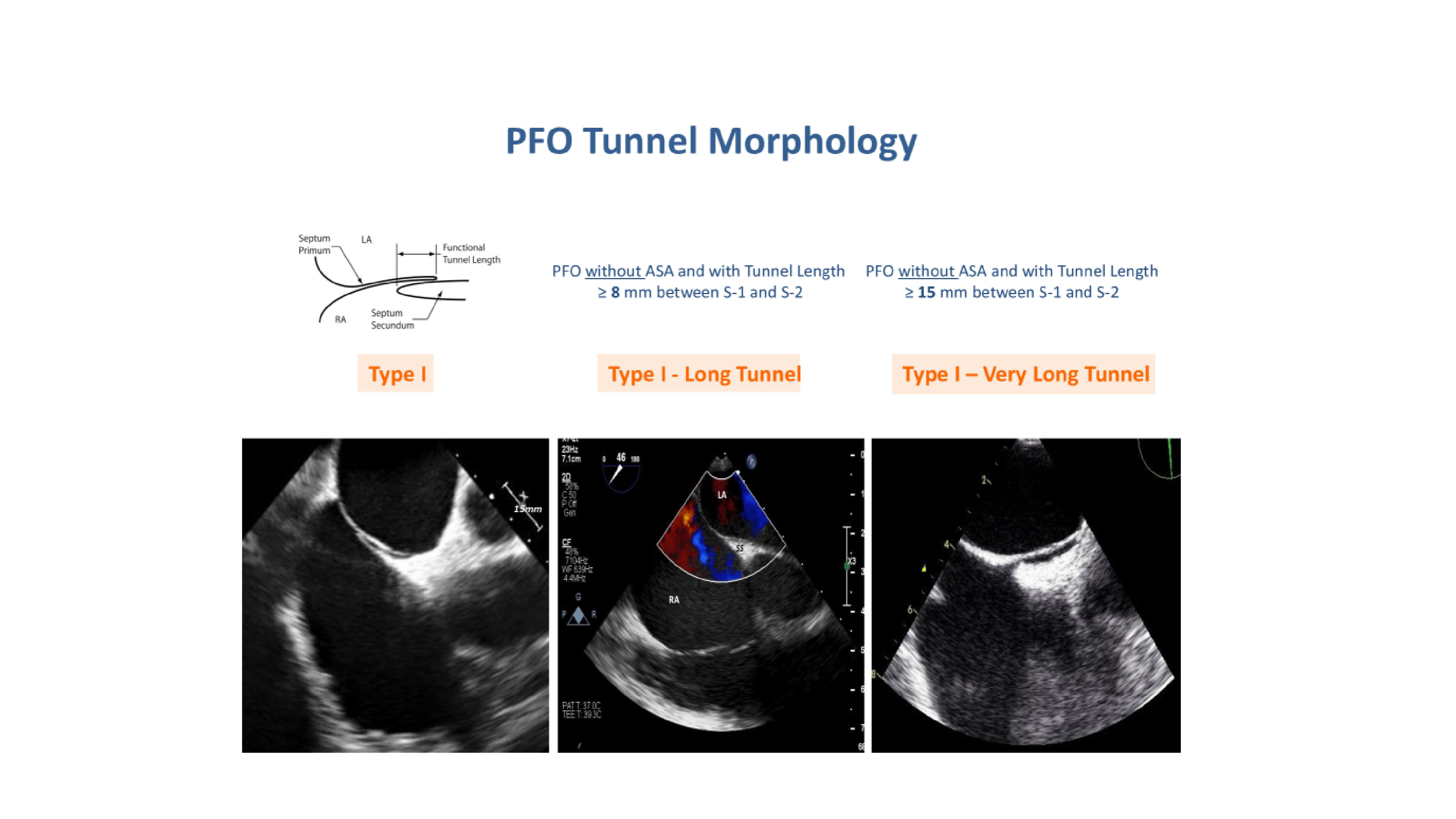

Supplement: Supplementary file 1 [file jcm-12-05788-s001.zip › Figure S2.jpeg]

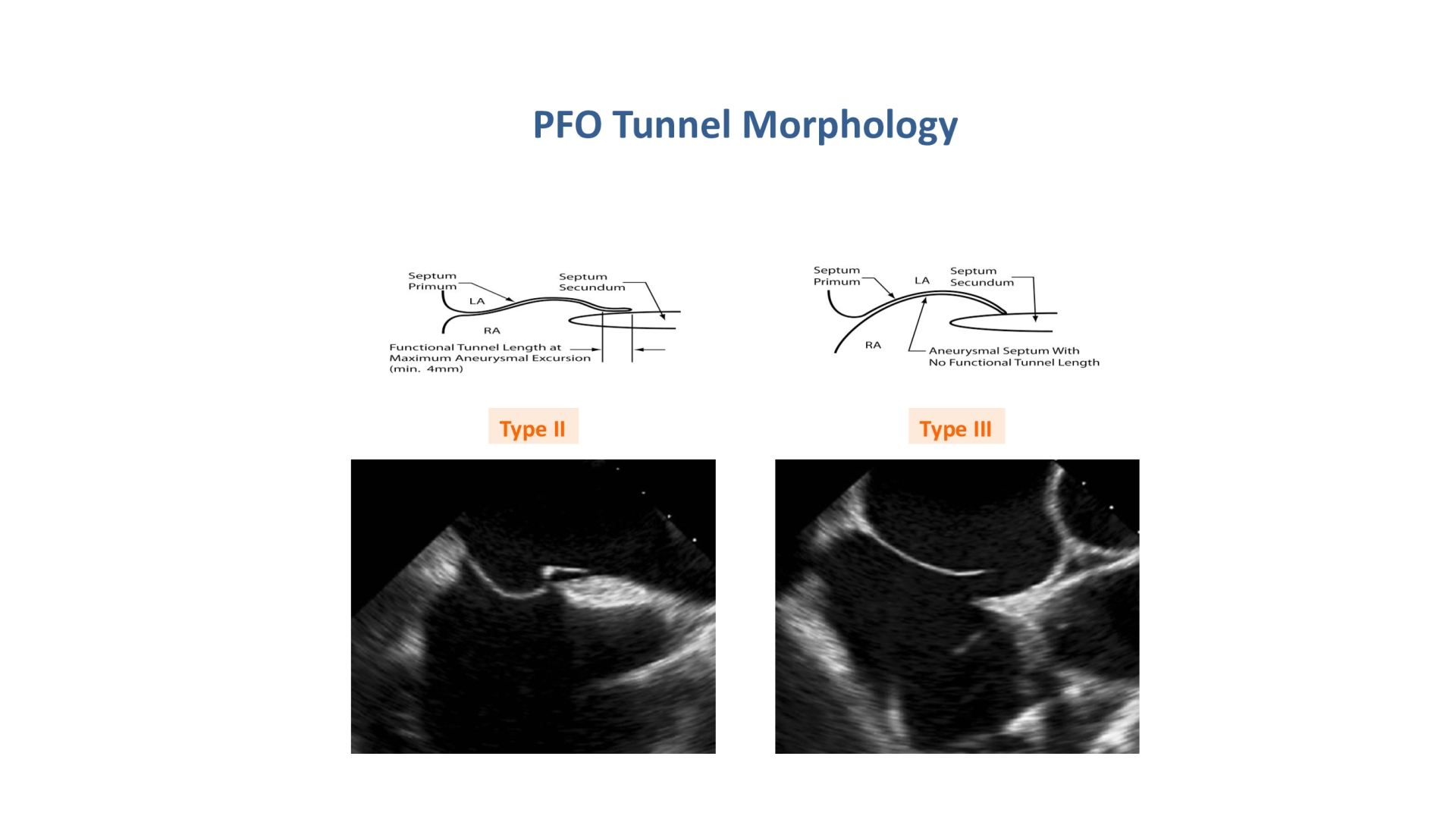

Supplement: Supplementary file 1 [file jcm-12-05788-s001.zip › Figure S3.jpeg]
